# Successful Stem Cell Transplantation in a Patient with Pretransplant Hepatic Inflammatory Pseudotumour

**DOI:** 10.1155/2016/6801916

**Published:** 2016-11-28

**Authors:** Sanjay de Mel, Zarni Soe, Benjamin Wong, Ching Ching Ong, Lynette Teo, Lip Kun Tan

**Affiliations:** ^1^Department of Haematology-Oncology, National University Cancer Institute, National University Health System, 1E Kent Ridge Road, NUHS Tower Block Level 7, Singapore 119228; ^2^Department of Pathology, National University Health System, 1E Kent Ridge Road, Singapore 119228; ^3^Department of Diagnostic Imaging, National University Health System, 1E Kent Ridge Road, Singapore 119228; ^4^Departments of Laboratory Medicine and Haematology-Oncology, National University Cancer Institute, National University Health System, 1E Kent Ridge Road, NUHS Tower Block Level 7, Singapore 119228

## Abstract

Inflammatory pseudotumours (IPT) are rare benign neoplasms of unknown aetiology. We present a case of hepatic IPT which was incidentally discovered in a patient with relapsed B-acute lymphoblastic leukaemia (B-ALL) undergoing pretransplant workup. After investigation to exclude an infective cause she underwent a reduced intensity conditioning stem cell transplant (SCT) successfully and currently remains well and in remission. On repeat liver MRI after SCT, the IPT was seen to be resolving. To the best of our knowledge this is the first report of an adult patient with hepatic IPT successfully undergoing SCT. The reduction in size of the IPT after SCT also suggests an inflammatory rather than an infective aetiology for IPT.

## 1. Background

IPT are rare benign tumours which can mimic malignant neoplasms both clinically and radiologically. Historically, the terminology used to describe IPT has been diverse, mainly due to variable histological findings. It was first described in 1939 by Brunn and the term IPT was first used by Umiker and Iverson in 1954 [1]. It has been reported to be found in many different sites including the lungs, orbit, spinal cord, gastrointestinal tract, spleen, pancreas, kidney, adrenal gland, bladder, thyroid, and the retroperitoneal region [2].

The first case of IPT of the liver was described by Pack and Baker in 1953 [3]. These patients have been reported to present with fever, abdominal pain, and jaundice, sometimes leading to biliary obstruction, portal hypertension, cirrhosis, and eventually hepatic failure [4]. The key histologic finding in IPT is proliferation of spindled myofibroblast cells mixed with variable amounts of reactive inflammatory cells [4].

Radiological features of IPT are also variable and nonspecific, possibly because of significant fibrosis and cellular infiltration [5]. Ultrasound can show hypo- or hyperechoic lesions with either ill-defined or well circumscribed borders [6]. These lesions often have increased vascularity during color or power Doppler examinations. CT findings are also diverse, with low, equal, or high attenuation compared with surrounding tissue having been described [6]. On MRI, IPT usually shows low signal intensity on both T1- and T2-weighted images, which may reflect the fibrotic nature of these lesions [7]. Contrast enhanced CT and MRI may show a homogenous or heterogeneous lesion. Delayed imaging often shows increasing enhancement due to the presence of fibrosis [5].

More than a hundred cases of IPT have been reported. Their aetiology is largely unknown; however, they have been postulated to be associated with inflammation, trauma, surgery, or an autoimmune process [8–10]. Treatment options remain experimental. IPT have been reported to be successfully treated by surgery, chemotherapy, and immunosuppressive therapy with rituximab in a case of orbital IPT [10–12]. There have been six cases of IPT diagnosed in patients after haematopoietic stem cell transplant (SCT) and one case of hepatic IPT in a pediatric patient before SCT with severe congenital neutropenia [13, 14]. To the best of our knowledge, ours is the first report of hepatic IPT in an adult who was due to undergo SCT.

## 2. Case Report

The patient was a 25-year-old lady diagnosed with standard risk B-ALL in October 2010. She was treated with the MASPORE (Malaysia-Singapore ALL study) intermediate risk protocol. Although she did not achieve a morphologic remission after her induction therapy, the MASPORE protocol was continued and she achieved a morphologic remission as well as flow cytometric minimal residual disease (MRD) negativity in December 2012. Unfortunately her ALL relapsed in August 2014. She did not respond to the Hyper-CVAD regimen but achieved a complete remission after salvage with the FLAG-Ida protocol in November 2014. Her treatment with FLAG-Ida was complicated by neutropenic sepsis and* Escherichia coli* (*E. coli*) bacteremia which was treated with intravenous meropenem for 14 days.

She was planned for SCT from a matched unrelated donor. A routine pre-SCT transthoracic echocardiogram showed a dilated right ventricle. A magnetic resonance imaging (MRI) scan of the heart was therefore performed to further investigate the right heart pathology; this showed no significant cardiac pathology but incidentally detected multiple, bilobar hepatic lesions.

**Figure 1:**
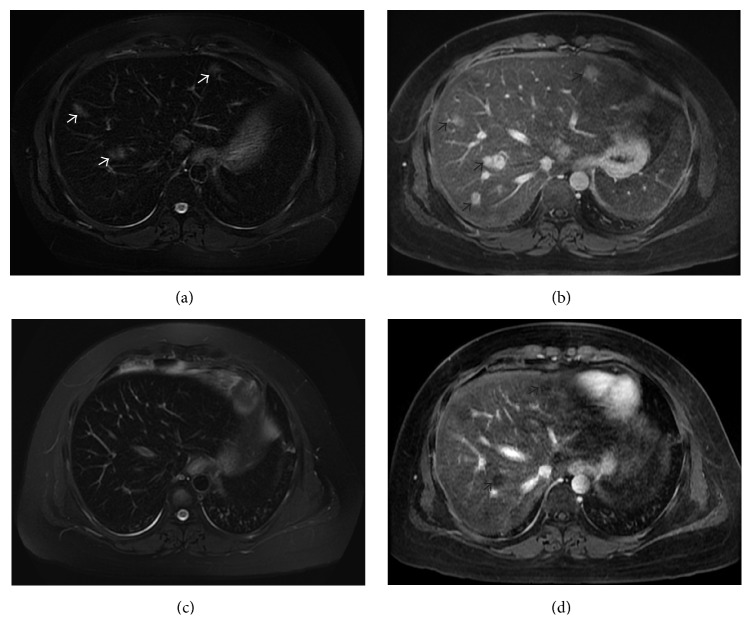
(a, b) Baseline MRI. (a) Axial T2-weighted fat saturated (T2W-FS) image shows faintly hyperintense lesions in the liver (white arrows). The liver parenchyma is hypointense and shows reduced signal in keeping with iron loading. (b) Axial delayed postcontrast T1W images show persistent delayed enhancement in the lesions (black arrows). (c, d) MRI after treatment. (c) Axial T2W-FS image shows that the lesions are isointense to the rest of the liver parenchyma which is hypointense (due to iron loading). The lesions are no longer hyperintense on the T2W-FS image. (d) Axial delayed postcontrast T1W images show that the lesions no longer show any appreciable enhancement.

An MRI scan of the liver was therefore performed which showed multiple lesions of varying sizes throughout both lobes of the liver (Figure 1). These lesions had ill-defined margins and showed hyperintense signal on the T2-weighted fat-saturated (T2W-FS) images. They showed a target appearance, with a central core of hyperintensity and a hypointense rim on the precontrast T1-weighted (T1W) images. The lesions showed early (mainly central) arterial enhancement, with persistent enhancement on the delayed postcontrast images. The patient had no abdominal pain, icterus, or fever. Abdominal examination revealed no tenderness or clinically detectable hepatomegaly. Her liver function tests (LFT) were normal.

**Figure 2:**
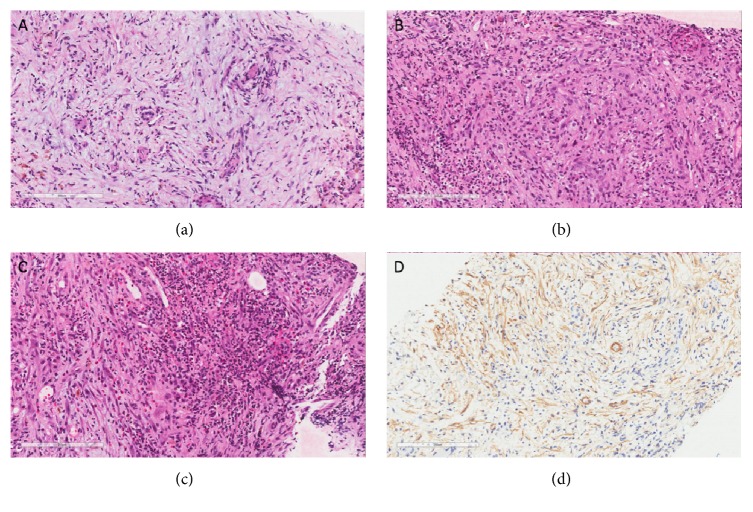
Pseudotumour histology. The H&E images (a, b, c) show a loose proliferation of fibrous tissue, accompanied by a mixed acute and chronic inflammatory infiltrate, showing entrapment of residual bile ducts and hepatic artery branches. The immunohistochemistry (d) test for smooth-muscle actin shows reactivity in spindle cells, consistent with a proliferation of myofibroblasts, as might be seen in reactive fibrosis or an inflammatory pseudotumour.

An ultrasound-guided liver biopsy was performed: this showed an extensive zone of cellular fibrosis with a mixed acute and chronic inflammatory infiltrate composed of eosinophils and aggregates of neutrophils (Figure 2). The zone of fibrosis merged into one of many markedly expanded portal tracts which showed oedema with a mild mixed inflammatory infiltrate. The spindle cells showed immunohistochemical reactivity with smooth-muscle actin (SMA), confirming myofibroblastic differentiation. This biopsy also showed two slender nonbranching fungal hyphae, resembling* Candida*. However, a repeat biopsy showed only cellular fibrosis accompanied by inflammatory infiltrate, with no evidence of fungal organisms. It was concluded that the fungi in the first biopsy were not of clinical significance (and were possibly a contaminant).

A decision was made to proceed with SCT from a matched unrelated donor using reduced intensity conditioning (RIC): fludarabine 30 mg/m^2^ for 4 days and busulfan 3.2 mg/kg for 3 days. Her graft versus host disease (GVHD) prophylaxis regimen included thymoglobulin (ATG), methotrexate, and tacrolimus. ATG was dosed at 0.5 mg/kg on D-3, 1.5 mg/kg on D-2, and 2.5 mg/kg on D-1 while methotrexate was given intravenously at 15 mg/m^2^ on D+1 followed by 10 mg/m^2^ on D+3, D+6, and D+11. Tacrolimus was commenced on D-3 aiming for a trough level of 8–15 *µ*g/L. No dose adjustments to her chemotherapy were made as her LFT were normal at the point of conditioning. The CD 34+ cell dose infused was 2.76 × 10^6^/kg. No prophylaxis for venoocclusive disease (VOD) was given and her LFT remained normal throughout the conditioning and recovery phases of the SCT. Apart from an episode of neutropenic fever, she had an uneventful post-stem cell infusion period with neutrophil engraftment occurring on D+26. A repeat MRI scan of the liver three months after SCT showed that the liver lesions were smaller in size and no longer hyperintense, suggesting some resolution of the inflammatory changes (Figure 1). She had no VOD or hepatic GVHD; she currently remains well and in remission with mild GVHD of the skin and a donor chimerism (CD3 lineage specific) of 98%.

## 3. Conclusions

The differential diagnoses for multiple hepatic lesions include a variety of infective, inflammatory, and neoplastic disorders. Pyogenic liver abscesses and disseminated fungal infection were strong considerations for our patient, especially given that she had* E. coli* bacteremia after her salvage chemotherapy. Her blood cultures were negative for bacteria and fungus while urine cultures and a chest radiograph were unremarkable.

Fangusaro et al. reported the first cases of IPT after SCT in two pediatric patients: one hepatic and one oesophageal, both treated by surgical resection [15]. All of the six reported cases of IPT after SCT were treated with surgical resection and the aetiology was thought to be SCT related immunosuppression and prior chemotherapy [13]. In contrast, a case of retroorbital IPT in a patient with systemic lupus erythematosus was successfully treated with rituximab [10]. The only reported case of IPT in a pre-SCT patient was a case of hepatic IPT in a pediatric patient with severe congenital neutropenia. Interestingly her IPT responded to granulocyte transfusion after which the SCT was successfully performed [14].

The decision of proceeding with SCT in our patient was difficult as an infective or neoplastic cause had to be excluded before subjecting her to the profound immunosuppression of SCT. The improvement of radiologic findings after SCT in our patient suggests an autoimmune or inflammatory aetiology for IPT rather than an infective cause. Another possibility is whether granulocyte colony stimulating factor (GCSF) may have contributed to the IPT; she was given GCSF following her treatment with FLAF-Ida. As she had no liver imaging before her course of FLAG-Ida we cannot be certain if the IPT was already present before her salvage therapy. Our case suggests that successful RIC SCT is possible in adult patients with IPT provided infective causes are excluded. Further studies are however required to elucidate the biology and natural history of these rare tumours.

## Abbreviations

IPT: Inflammatory pseudotumour
B-ALL: B-acute lymphoblastic leukaemia
SCT: Stem cell transplant
MRI: Magnetic resonance imaging
*E. coli*: *Escherichia coli*
MRD: Minimal residual disease
VOD: Venoocclusive disease
GCSF: Granulocyte colony stimulating factor
GVHD: Graft versus host disease.
